# Highly Selective Fluorescent Sensors: Polyethylenimine Derivatives of Triphenylamine and Coumarin for GTP and ATP Interaction via Fluorescence Lifetime Imaging Microscopy

**DOI:** 10.1021/acsapm.3c00834

**Published:** 2023-07-11

**Authors:** Estefanía Delgado-Pinar, Matilde Medeiros, Telma Costa, J. Sérgio Seixas de Melo

**Affiliations:** † CQC-IMS, Department of Chemistry, University of Coimbra, Rua Larga, Coimbra 3004-535, Portugal; ‡ Instituto de Ciencia, Molecular, Departamento de Química Inorgánica, Universidad de Valencia, C/Catedrático José Beltrán 2, Paterna 46980, Spain

**Keywords:** PEI, fluorescence, AIE, adenosine-5′-triphosphate (ATP), guanosine-5′-triphosphate (GTP), FLIM imaging

## Abstract

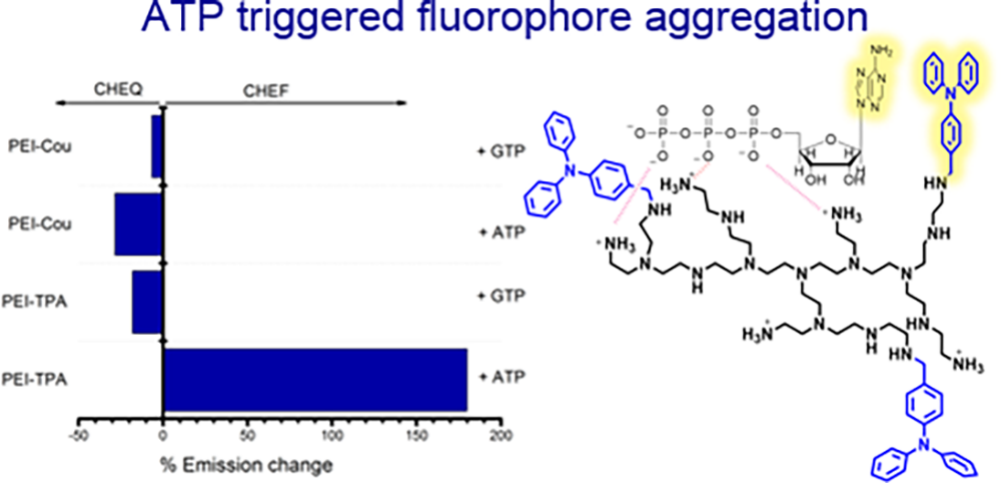

Chemical derivatives of polyethylenimine (PEI) receptors with either triphenylamine (TPA) or 7-hydroxy-4-methyl-coumarin (Cou) form stable complexes with adenine and guanine nucleotides in water. The host–guest complex modulation is found to be based on noncovalent molecular interactions such as π–π stacking and hydrogen bonding, which are dependent on the aromatic moieties attached to the polyaminic (PEI) backbone. **PEI-TPA** acts as a chemosensor with a recognition driving force based on aggregation-induced emission (AIE), involving π–π interaction between the nucleic base and TPA. It detects GTP by a chelation enhancement quenching effect of fluorescence (CHEQ) with a measured logarithm stability constant, log β = 7.7. By varying the chemical characteristics of the fluorophore, as in the **PEI-Cou** system, the driving force for recognition changes from a π–π interaction to an electrostatic interaction. The coumarin derivative detects ATP with a log β value one order of magnitude higher than that for GTP, allowing for the selective recognition of the two nucleotides in a 100% aqueous solution. Furthermore, fluorescence lifetime imaging microscopy (FLIM) allows for a correlation between the selectivity of **PEI-TPA** toward nucleotides and the morphology of the structures formed upon ATP and GTP recognition. This study offers valuable insights into the design of receptors for the selective recognition of nucleotides in water.

## Introduction

Nucleotides form the fundamental units of nucleic acids, with adenosine-5′-triphosphate (ATP) and guanosine-5′-triphosphate (GTP) recognized for their critical roles as universal energy sources in protein synthesis. ATP serves as the universal energy currency in living cells,^1^ participating in various biological processes including ion channels,^2^ neurotransmission,^3^ and muscle contraction.^4^ Deviations in ATP levels are linked to several pathological conditions, including lymphocytic leukemia,^5^ Parkinson’s disease,^6^ and cardiovascular disease.^7^ GTP plays a role in RNA synthesis, the citric acid cycle, and the stacked guanine tetrads in G-quadruplex, a target in antitumor therapies.^8^ As a result, developing molecular probes with high selectivity and sensitivity, fast response times, and tissue imaging capability is crucial for investigating the role of these nucleotides in biology and diagnosing diseases.

There is a fairly wide range of fluorescent probes that have demonstrated their effectiveness in the detection of different nucleotides, such as ATP, over the corresponding mono- and diphosphate nucleotides or GTP.^9−11^ They are mainly based on organic dyes,^12^ polymers,^13^ and nanoparticles.^14^ However, designing molecules that can bind selectively to ATP or GTP in 100% aqueous solution still constitutes a significant challenge, as these two nucleotides are very similar in size, structure, and charge. Moreover, the receptors must function in aqueous solutions at physiological pH, and distinguishing between anions is another challenge, as the synthetic receptors must compete with water molecules (and metal ion cofactors) for the negatively charged phosphate groups.^15^ Therefore, multifunctional receptors that incorporate electronic and steric characteristics to develop different binding contributions, such as electrostatic charge–charge attractions, hydrogen-bond formation, and hydrophobic or π–π interactions, are usually included in the design.^16,17^

In this work, we have developed branched polyethylenimine (PEI) derivatives with aromatic moieties as suitable receptors for ATP and GTP. Previous studies have shown that PEIs react readily with negative and metal ions through neutralization or complexation.^18,19^ PEIs have also been shown to be promising gene or drug carriers for in vitro and in vivo applications,^20−23^ and they exhibit strong buffering ability.^24^ Although the significant cationic charges on the polymer surface can induce toxic effects, this drawback can be mitigated by reducing the positive charge density.^25^ The design of receptors herein described has taken into account this particular aspect. The PEI receptors developed in this study feature repeating cationic amphiphilic structures that interact with the anionic guest through positively charged ammonium groups. Nonprotonated amine groups interact via hydrogen bonding with the ethylene backbone, serving as hydrophobic groups. Aromatic fragments incorporated into the backbone establish stacking interactions with the nucleotide bases and promote hydrophobic interactions.^26^ The chosen fluorophores incorporated in the cationic polymer also serve as luminescent probes for detecting and quantifying the interaction, with fluorescence emission being a useful signaling property due to its simplicity, high sensitivity, good selectivity, and real-time reaction monitoring. In the present study, we report two PEI systems modified with triphenylamine or 7-hydroxy-4-methyl-coumarin, capable of recognizing ATP or GTP to different extents in 100% aqueous solution (pH = 7.4, 10 mM Tris buffer).

## Experimental Section

### Materials and Methods

PEI (Aldrich Cat. No. 40871-9, average *M*_w_ = 800 g·mol^–1^ by LS, average *M*_n_ = 600 g·mol^–1^), 4-(*N*,*N*-diphenylamino)benzaldehyde, 3-chloro-7-hydroxy-4-methylcoumarin, and 7-hydroxy-4-methylcoumarin were obtained from commercial sources and used as received.

Water was twice distilled and passed through a Millipore apparatus. All solvents (spectroscopic or equivalent grade) were used without further purification. The pH values were measured with a 3510 Jenway pH meter, and adjustments to the hydrogen ion concentration of the solutions were made with diluted HClO_4_ and NaOH solutions.

^1^H NMR and ^13^C NMR spectra were recorded using a Bruker-AMX with an operating frequency of 400 and 101 MHz, respectively. ^13^C NMR spectra were performed at 25 °C using D_2_O as a solvent. NMR spectra were processed using MestReNova Software (Mestrelab Research, Spain). PEI and substituted PEI samples were first dried in vacuum for 24 h, then freshly prepared in D_2_O, and sent to spectra acquisition. Inversely gated decoupling pulse sequences were used for the measurement of the ^13^C NMR to avoid the influence of the Nuclear Overhauser effect on the signal intensities; thus, they could be used to unravel its architecture. Quantitative inverse-gated ^13^C-spectra were acquired using a 12 μs ^13^C-detection pulse, 1 s acquisition time, and 4 s recycle delay.

Absorption and fluorescence spectra were recorded with a Shimadzu UV-2100 spectrophotometer and a Horiba-Fluoromax spectrofluorimeter, respectively. All the fluorescence spectra were corrected for the wavelength response of the system. The absorption of the solutions was kept under 0.1 at the excitation wavelength to avoid the inner filter effects.^27^

Molar extinction coefficients were measured from the obtained slope from the plot of the optical density of the measured solutions as a function of concentration, according to the Beer–Lambert law, giving the values of 5020 M^–1^ cm^–1^ for **TPA-Cou** and 86,770 M^–1^ cm^–1^ for **PEI-TPA**. Fresh solutions were always prepared to avoid nondesirable aggregate particles formation. The concentration of the samples ranged from 0.2 to 4 μM depending on whether protonation (100% aqueous solution) or nucleotide (buffer solution) titrations were performed. A stock solution of ATP and GTP, at a concentration of 0.5 mM, was prepared in a buffer solution of Tris 10 mM and KCl 50 mM at physiological pH (7.4). Finally, aliquots of the proper nucleotide stock solution were added to the solution containing the PEI derivative. Following each addition, the pH was registered, and no appreciable value changes were observed.

For the determination of complex formation constants (β values), the spectrophotometric data were fitted with the program HypSpec.^28^

Fluorescence lifetime images were collected with a Becker and Hickl (GmbH) DCS-120 Confocal FLIM system.^29^ The system is equipped with a TCSPC-System module (SPC-150N), a NIKON Ti2-U inverted optical microscope, controlled by a galvo-drive unit (Becker and Hickl GDA-121), and a hybrid GaAsP photodetector (300–720 nm detection range), controlled by a DCC-100 detector controller card. The two objectives, 20× (CFI Plan Achromat 20×/0.40/1.20) and 40× (CFI Plan-Achromat 40×/0.65/0.56), were used. The DCS-120 confocal microscope system is equipped with a polarizing beam splitter. The excitation source is a picosecond diode laser of 375 nm wavelength (bh BDL series lasers) working in a pulsed mode (repetition rate: 80 MHz). The IRF of the system is found to be less than 100 ps. The total laser power at the sample was set to 40% of the maximum value, and the collected emission passed through a 1 mm pinhole, a long pass filter 390LP, and a band pass filter 445 nm. The FLIM images were scanned and recorded at a resolution of 512 × 512 pixels using the “FIFO imaging” mode of the SPC-150N modules. Data analysis was performed through SPCImage NG data analysis software. The decay curves were fitted using the maximum-likelihood algorithm (or maximum-likelihood estimation, MLE) fitting method, in each pixel. A drop of 20 μL sample solution was placed on top of a glass slide and was covered with a coverslip. The measurements were performed by placing the inverted slide on the microscope stage.

Light scattering measurements were performed on an ALV spectrometer consisting of a goniometer and an ALV-5004 multiple-tau full-digital correlator (320 channels), which allows measurements over an angular range from 301 to 1501. A He–Ne laser (wavelength of 632.8 nm) is used as the light source. Samples were independently filtered through membrane filters with a pore size of 0.45 mm (LCR Millipore).

### Synthesis and Characterization of Substituted PEI Derivatives

For the synthesis of the TPA derivative (**PEI-TPA**), branched PEI (1 g, 1.25 mmol) was dissolved in 40 mL of anhydrous ethanol, followed by dropwise addition of 4-(*N*,*N*-diphenylamino)benzaldehyde (0.27 g, 1 mmol) dissolved in 20 mL of CH_2_Cl_2_. The reaction was stirred at room temperature for 12 h under a nitrogen atmosphere. Then, a 10-fold excess of NaBH_4_ was added portion-wise. The reaction was then stirred for 1 h. The solvent was subsequently evaporated to dryness. The residue was treated with CH_2_Cl_2_ and repeatedly extracted with water (3 × 40 mL). The aqueous phase was dried, and the product was dissolved in ethanol. Then, the solvent was evaporated to dryness to give an oil (1.63 g, 83%).^1^H NMR (400 MHz, D_2_O): δ = 8.19–8.04 (m, 6H), 7.23 (m, 12H), 7.08–7.01 (m, 24H), 3.36–2.12 (m, 147H). ^13^C NMR (101 MHz, D_2_O): δ = 165.6, 147.4, 129.4, 123.7, 51.7, 45.6, 37.2.

**PEI-Cou** was synthetized following a similar procedure. Branched PEI (1 g, 1.25 mmol) was dissolved in 40 mL of anhydrous ethanol, followed by dropwise addition of 3-chloro-7-hydroxy-4-methylcoumarin (0.2 g, 0.62 mmol) dissolved in 20 mL of CH_2_Cl_2_. The resulting mixture was stirred for 24 h at 50 °C, and then, the solvent was evaporated to dryness. The residue was purified through alumina column chromatography using a mixture of CH_2_Cl_2_:acetone (50:50) as the eluent to obtain the desired compound (0.57 g, 47%). ^1^H NMR (400 MHz, D_2_O): δ = 7.97 (bd, 1H), 7.26 (bd, 1H), 6.56 (bs, 1H), 3.33–2.56 (m, 100 H). ^13^C NMR (101 MHz, D_2_O): δ = 164.5, 162.3, 156.2, 139.1, 124.6, 121.1, 118.3, 117.4, 98.8, 53.2, 52.5, 50.7, 48.7, 47.2, 45.4, 39.0, 37.4.

## Results and Discussion

### Characterization of PEI

To determine the relative proportion of primary, secondary, and tertiary amines in the starting commercial PEI, ^13^C NMR analysis was performed (Figure S1), and the signal assignment was carried out following the reported literature.^30^ In branched polyethylenimine, multiple building blocks are present, as shown in Figure S2. Therefore, inverse-gated ^13^C can be utilized to unravel the architecture of the polymer. The ratio of primary, secondary, and tertiary amines in commercial branched PEIs is a crucial parameter that affects not only technical performance but also toxicity.^31^ The unmodified PEI initially contained 44, 26, and 30% of primary (1), secondary (2), and tertiary (3) amines, respectively, as determined by equation Seq1 (in SI).^32^ Additionally, it is possible to determine the number of amine groups on one PEI unit and, combined with the previous information, the number of primary, secondary, and tertiary amines (see Seq2 in SI). These data can also be presented as the relative ratio of linear to branched structures, calculated from the carbon NMR integrals of secondary (2) to tertiary (3) amines. For the commercial PEI, the obtained ratio is 0.86, indicating that tertiary nitrogen atoms are situated close to another branching point, reflecting the high degree of branching of the PEI.

### Synthesis and Characterization of Substituted PEI

**Scheme 1:**
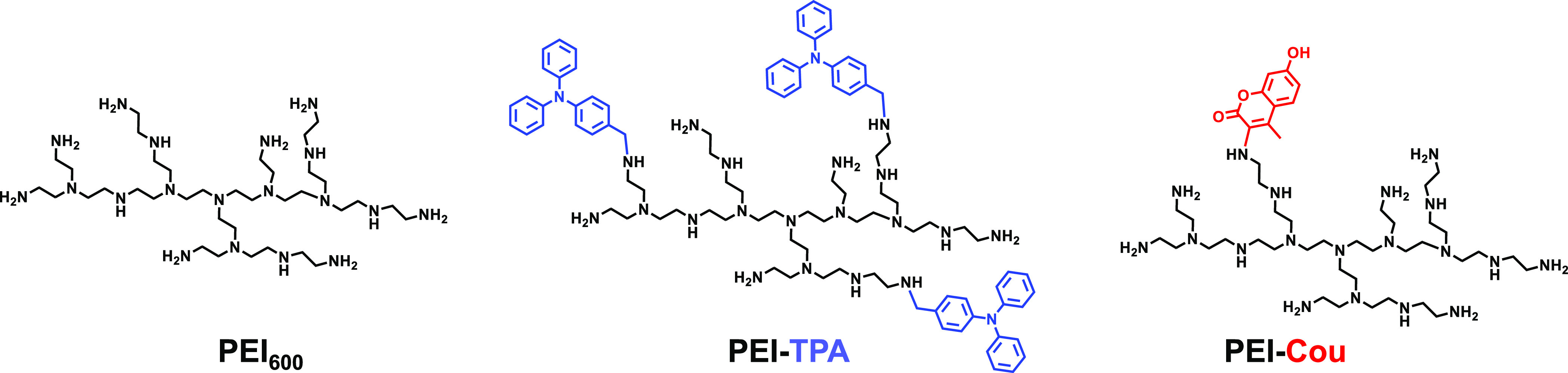
Chemical Structures and Acronyms of the Studied PEI Derivatives

Two PEI derivatives, **PEI-TPA** and **PEI-Cou**, were obtained by making use of different amine groups present in PEI (Scheme 1). The incorporation of the triphenylamine moiety (TPA) was accomplished by reacting a primary amine with an aldehyde to form the imine through a Schiff’s base reaction, followed by reduction to the corresponding amine (**PEI-TPA**). The incorporation of 7-hydroxy-4-methylcoumarin, through reaction with 3-chloro-7-hydroxy-4-methylcoumarin, was achieved by direct substitution in the primary amines present in PEI (**PEI-Cou**).

The inverse-gated ^13^C NMR spectrum of **PEI-TPA** (Figure S3) revealed signals from the polymer as well as additional signals from the triphenylamine. The binding of TPA was confirmed by the presence of a signal at 55 ppm from the benzylic methylene group and the absence of a signal at 65 ppm from the benzylic alcohol that would be obtained after the reduction of the TPA carboxyaldehyde with sodium borohydride. Moreover, it is possible to observe the reduction in intensity area of the signals related to primary amines (ethylene chains close to a primary amine, see Figure S1) since primary amines are the functional groups that react with the aldehyde. Finally, it is possible to obtain the aromatic signals coming from the TPA in their correct chemical shift displacements (see Figure S4 that shows the ^1^H NMR for the **PEI-TPA** system).

The binding of **PEI-Cou** NMR spectra is supported by a signal at 139 ppm indicating the binding of the coumarin moiety to the polyamine backbone in the 3-position through aromatic substitution of the coumarin structure (Figures S5 and S6 for the ^13^C NMR and ^1^H NMR, respectively).

**Figure 1:**
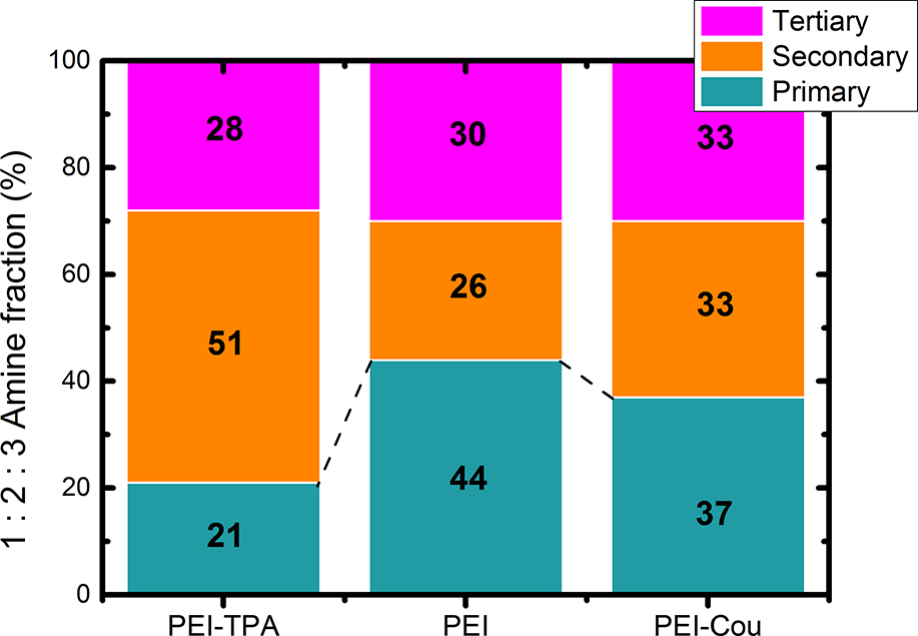
Amine (primary, 1, secondary, 2, and tertiary, 3) distributions of PEI and functionalized PEIs analyzed by ^13^C NMR.

The results indicate that functionalization selectively occurs with primary amines, converting them into secondary amines. Liquid-phase ^13^C NMR was used to characterize the amine state distributions in PEI and its derivatives (Figure 1). The degree of substitution was found to be dependent on the system, with 50% of primary (1) amines modified in the case of **PEI-TPA** and only 16% modified in the case of **PEI-Cou**. The proportion of primary amines decreased in both compounds after functionalization.

### Spectral and Photophysical Behavior of PEI Derivatives in Water

#### Absorption and Steady-State Fluorescence

The interaction of PEI derivatives with ATP and GTP was first performed by examining the dependence of the absorption and fluorescence spectra of **PEI-TPA** and **PEI-Cou** with the pH.

**Figure 2:**
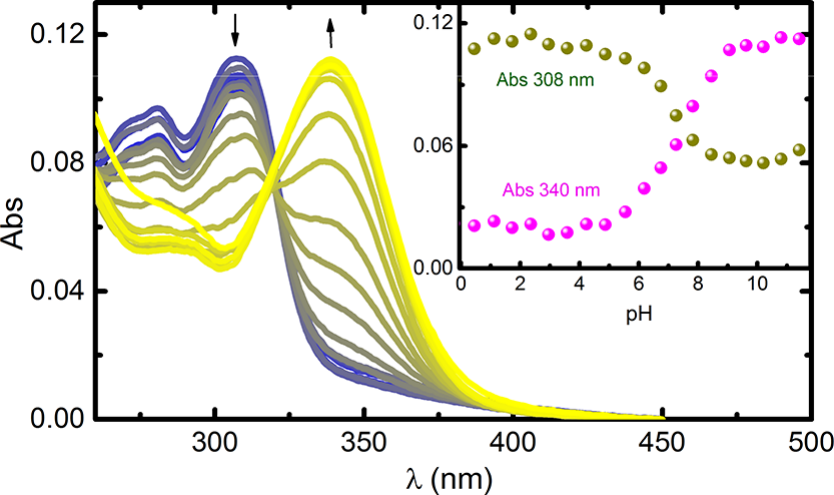
Absorption spectra of **PEI-Cou** recorded at 298.1 ± 0.1 K as a function of pH. The inset displays the dependence of the absorbance with the pH followed at 308 nm (blue) and 340 nm (yellow). [**PEI-Cou**] = 1.7 × 10^–5^ M.

Figure 2 displays the UV–visible absorption spectra of **PEI-Cou** as a function of pH. In an acidic aqueous solution, **PEI-Cou** exhibits a maximum absorption at 310 nm, corresponding to the neutral form (N) of 7-hydroxy-4-methylcoumarin (Cou).^33,34^ As the pH increases, the absorbance at 310 nm decreases and a new absorption maximum appears at 340 nm, corresponding to the anionic form (A) of Cou. An isosbestic point is observed at 320 nm, which reflects the acid–base equilibrium involving N and A.

From the titration curve, a p*K*_a_ value of 7.4 was obtained, for **PEI-Cou**, which is similar to the p*K*_a_ values of 7-hydroxy-4-methylcoumarin and other related coumarins with equivalent substitutions; e.g., p*K*_a_ = 7.7 for 3-[2-(diethylamino)ethyl]-7-hydroxy-4-methylcoumarin or p*K*_a_ = 7.2 for 3-chloro-4-methylumbelliferone (3Cl4MU).^33,34^ This indicates that the incorporation of the coumarin moiety into the PEI structure did not change its acid–base properties in the ground state. However, the steady-state fluorescence behavior was found to be different, with the presence of an additional species and a much lower excited state p*K*_a_^*^ value.

**Figure 3:**
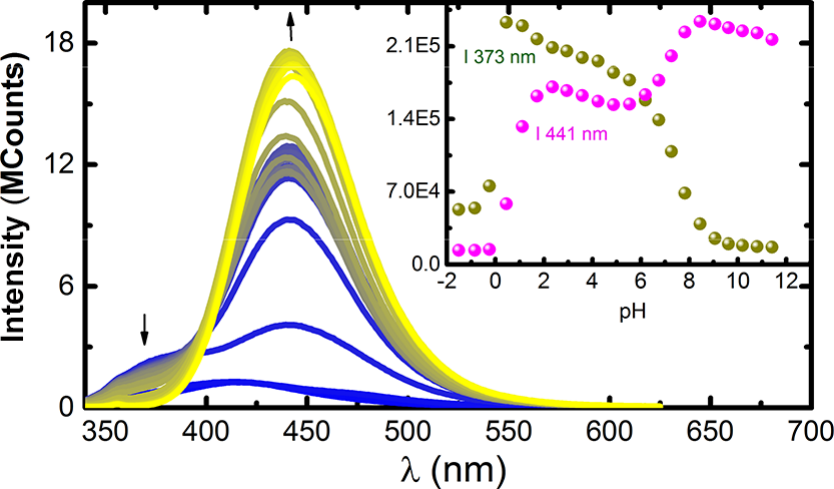
Fluorescence emission (λ_exc_ = 318 nm) spectra for **PEI-Cou** as a function of pH at *T* = 298 K. The inset shows the emission of **PEI-Cou** followed at 373 (blue) and 441 nm (yellow). [**PEI-Cou**] = 1.7 × 10^–5^ M. λ_exc_ = 318 nm.

The dependence of the fluorescence emission spectra of **PEI-Cou** with the pH was examined (Figure 3), and it revealed the emission of the neutral form, N* (with maxima at ca. 373 nm), at very low pH values. Interestingly, there was no evidence of the emission of the tautomeric form, T*, of 7-hydroxy-4-methylcoumarin (7H4MC), as is usually observed.^34^ This indicates that the A* ↔ T and N* ↔ T* equilibria present at very low pH values disappear,^35^ and the N* ↔ A* is now the only excited state equilibria present (see Figure S7 for comparing **PEI-Cou** and 7H4MC emission at acidic pHs).^36^ 7H4MC exhibits a single acid–base equilibrium with pK*_a_ = 0.6. However, **PEI-Cou** seems not to have the corresponding equilibria, with the formation of the tautomeric form, probably due to hydrogen bonding between the excited neutral form N* of 7H4MC with the positive charges of PEI. As the pH is increased, the anionic form, A* (with maxima at ca. 441 nm), begins to appear with the concomitant disappearance of N* (with maxima at ca. 373 nm). From the dependence with pH of the emission intensity of these two bands, two apparent excited state p*K*_a_^*^ values for the acid–base equilibria were calculated: p*K*_a,1_^*^ = 0.7 and p*K*_a,2_^*^ = 7.3. This behavior is very interesting since the literature shows shifting of the p*K*_a_^*^ value by encapsulation of the coumarin in cucurbituril macrocycles but not the existence of two excited state acid–base equilibria.^37,38^

The enhancement of the fluorescence at 441 nm (anionic, A*, emission) above pH 7 can be understood based on the acid–base equilibria of PEI influencing the photophysical behavior of the coumarin. Deprotonation of the amines present in the PEI skeleton favors the disruption of the intermolecular interactions such as hydrogen bonding and/or electrostatic interactions, responsible for the unusual presence of the neutral form (N*) in this pH range. After the deprotonation of the amines present in the polymer, a total disappearance of the neutral form and an increase of the anionic (A*) form of the coumarin are achieved.

**Figure 4:**
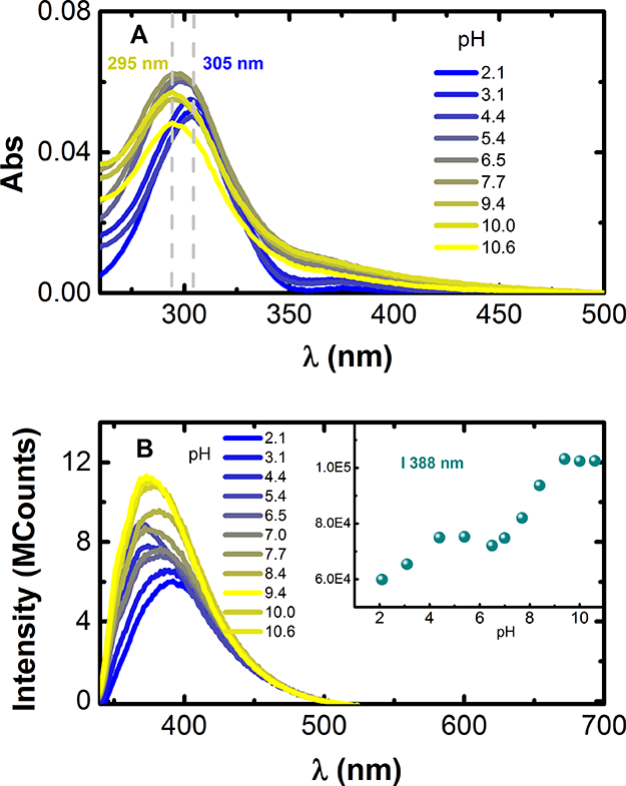
(A) Absorption and (B) fluorescence emission spectra B of **PEI-TPA** (λ_exc_ = 300 nm) at different pH values. [**PEI-TPA**] = 7.1 × 10^–7^ M.

Further investigation of **PEI-TPA** shows that the basicity of TPA amine is quite low, and therefore, it does not have any acid–base equilibria within the investigated pH range (pH = 2.5–11.0). Consequently, only the acid–base equilibria of PEI should be considered to explain the observed changes (PEI, p*K*_a1_ = 6 p*K*_a2_ = 9).^39^Figure 4 illustrates the changes in the absorption and fluorescence spectra of **PEI-TPA** with varying pH. As the secondary amine groups undergo continuous deprotonation, there is a blue shift in the wavelength absorption maxima of approximately 10 nm. In contrast, the deprotonation of the primary amines, which are not substituted with TPA, results in a slight decrease in absorption and an increase in the spectral baseline, suggesting the formation of very small aggregates within this pH range (Figure 4A).

In contrast, the occurrence of electrostatic repulsion between the positively charged backbone is eliminated upon PEI deprotonation, facilitating the formation of intermolecular interactions among the aromatic units. This, in turn, leads to the constraining of the intermolecular rotation of the TPA moiety through π–π interactions between TPA units, giving rise to the aggregation-induced emission (AIE) effect and a concurrent enhancement in fluorescence emission (Figure 4B).^40,41^ The observed changes in the fluorophore’s emission reveal the existence of two excited state acid–base equilibria (p*K*_a_^*^ = 3 and p*K*_a_^*^ = 8), which can be solely attributed to the polyamine backbone. The variations in the p*K*_a_^*^ values, in comparison to literature data,^39^ can be attributed to two factors: (i) structural modifications in PEI that may affect the basicity of the amine groups present in the backbone and (ii) possible cooperative shifting of p*K*_a_ by primary and secondary amines, resulting in a single acid–base equilibrium.^42^ It is important to note that in the PEI structure, protonation patterns are average situations and that the overall backbone structure may display the involvement, to some extent, of all the amine basic centers.

### Nucleotide Interaction with PEI-TPA and PEI-Cou

This study aimed to investigate nucleotide binding with **PEI-TPA** and **PEI-Cou** in an aqueous solution using spectroscopic (absorption and fluorescence) titrations. As a consequence of the PEI structure, electrostatic interactions between the positively charged PEI and negatively charged phosphate groups, present in the mononucleotides, would result in complex formation. Meanwhile, the interaction between the fluorophore units and nucleic bases will lead to the selective recognition of the nucleotides depending on which binding mode is more representative: H-bonds or π–π stacking.

**Figure 5:**
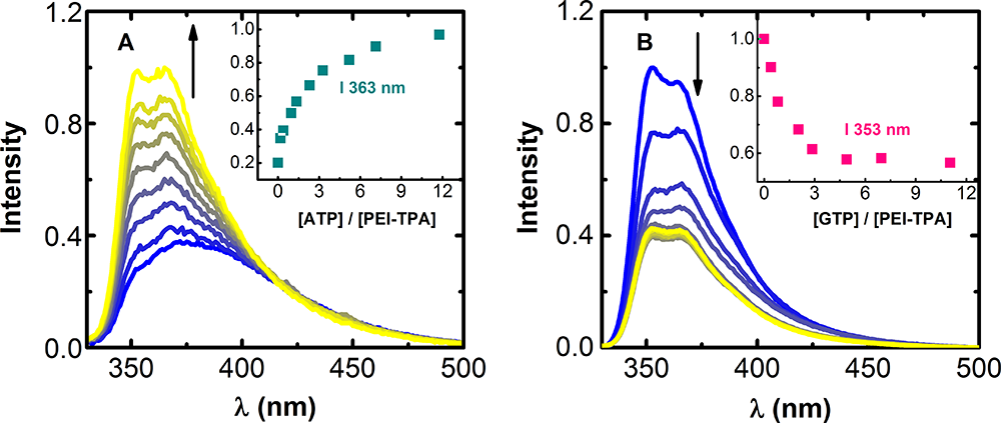
Normalized emission spectra resulting from the addition of (A) ATP and (B) GTP to an aqueous solution of **PEI-TPA** at pH = 7.4. Inset: changes in emission at maximum fluorescence upon the addition of an increasing amount of nucleotide. (A) [**PEI-TPA**] = 5.17 × 10^–7^ M and (B) [**PEI-TPA**] = 2.44 × 10^–7^ M. [ATP] = [GTP] from 0 to 6 × 10^–6^ M and λ_exc_ = 300 nm.

The results show that the addition of ATP to **PEI-TPA** gives rise to a significant change in its spectroscopic behavior, with an increase in the maximum emission intensity observed upon the recognition event (Figure 5A, see Figure S8A for absorption). Similarly, the addition of increasing amounts of GTP leads to a high quenching of the fluorescence of the TPA moiety (Figure 5B, see Figure S8B for absorption). Interestingly, the addition of ca. three equivalents of GTP completes the suppression of the emission, which corresponds to the quantity of TPA units present in the **PEI-TPA** system. These results indicate that the effect on the fluorescence emission is mainly influenced by π–π interactions between the TPA in **PEI-TPA** and the nucleic base (in ATP and GTP).^43,44^ Formation of small aggregates during the interaction between **PEI-TPA** with GTP, as seen by the enhancement of the absorbance at the red edge of the absorption spectrum, together with the appearance, and concomitant increase, of a band centered at 375 nm (Figure S8B) allowed easy discrimination between ATP and GTP at physiological pH.

The photophysical response observed by **PEI-TPA** can be primarily attributed to the ability of TPA, which is an extended aromatic molecule, to form π–π interactions with other aromatic moieties and, second, to the presence of protonated and/or deprotonated amines that facilitate intermolecular interactions, such as hydrogen bonding, between the polyamine chains and the phosphate groups, promoting the formation of small aggregates in the solution.^40^ When ATP is added to the solution, the propeller shape of the TPA moiety establishes π–π interactions that restrict the intramolecular rotation (RIR) of the TPA unit. This RIR restriction is responsible for the observed AIE effect.

On the other hand, due to the higher affinity of the TPA molecule toward GTP,^26,45^ or even toward oligonucleotide-rich G-quartet structures,^46^ the π–π interactions between TPA and guanine result in an effective stacking interaction with the fluorophore, and hence, in the fast formation of larger aggregates, as seen in Figure S8B. This leads to the CHEQ effect, typically seen in organic fluorophores when unstructured aggregates are present. The stability constants of the complexes formed in these systems were determined using spectrofluorimetry. Titration experiments were conducted in a buffer solution of Tris 10 and KCl 50 mM at physiological pH = 7.4. The results showed log β values of **PEI-TPA**-ATP = 6.1(1) and **PEI-TPA**-GTP = 7.7(2), indicating a higher preference for GTP than ATP. (The results of a selectivity experiment of **PEI-TPA** toward both nucleotides are shown in Figure S9.)

**Figure 6:**
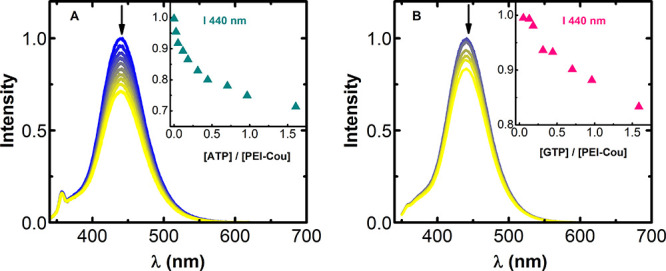
Normalized emission spectra resulting from the addition of (A) ATP and (B) GTP to an aqueous solution of **PEI-Cou** at pH = 7.4. Inset: changes in emission at maximum fluorescence upon the addition of an increasing amount of nucleotide. (A) [**PEI-Cou**] = 3.81 × 10^–6^ M and (B) [**PEI-Cou**] = 8.86 × 10^–6^ M. [ATP] = [GTP] from 0 to 6 × 10^–6^ M. λ_exc_ = 318 nm.

Regarding the sensing ability of the **PEI-Cou** system, it is worth noting that the difference in the **PEI-TPA** receptor is found in the sensing moiety. Spectroscopic titrations for **PEI-Cou** are shown in Figure 6 (UV–vis spectroscopic results are available in Figure S10). When GTP and ATP are added to the solution, the anionic form of the coumarin exhibits almost a complete emission quenching. Steady-state titrations were used to determine the stability constants of the complexes formed in these systems, resulting in log β values of 6.1(5) for **PEI-Cou**-ATP and 5.3(1) for **PEI-Cou**-GTP. This indicates a much higher preference for ATP than for GTP in this case.

**Scheme 2:**
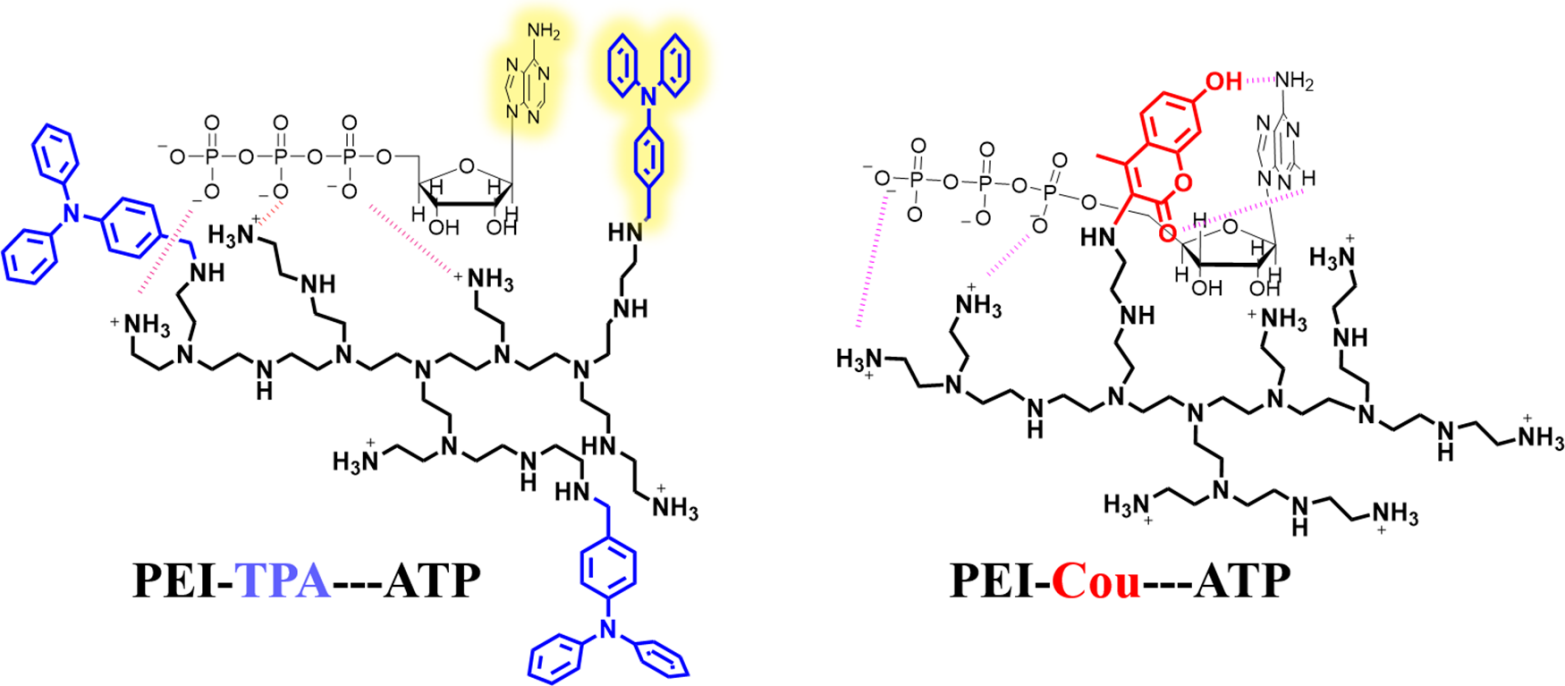
Schematic Illustration for the Proposed Mechanism for ATP Recognition with **PEI-TPA** and **PEI-COU**

Experimentally, the receptor based on coumarin (**PEI-Cou**) has a higher affinity for adenine than for guanine, indicating that the binding mode, between coumarin and the nucleotides, should be different than with triphenylamine. This can be due to the formation of a double hydrogen bond (one acting as a donor and the other as an acceptor) between the coumarin and the adenine in ATP, which is not possible with guanine in GTP. Indeed, with guanine, the formation of this type of hydrogen bond is precluded due to the presence of a carbonyl group at the position where a NH_2_ group exists in adenine. Therefore, with GTP, a hydrogen-bond acceptor group is present in this position instead of a donor group. We further propose that in **PEI-TPA**, AIE is the driving force for the recognition of the nucleotides, whereas in the case of **PEI-Cou**, electrostatic interactions determine the selectivity of the nucleotides (Scheme 2).

**Figure 7:**
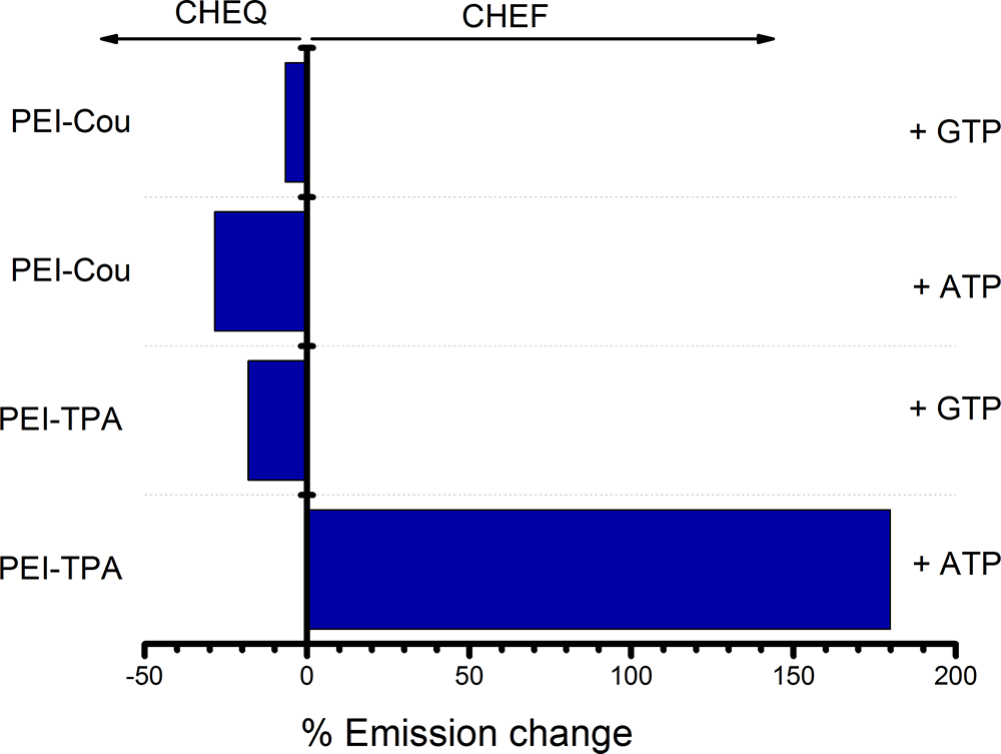
Bar diagram representation of the % response relative fluorescence intensity of **PEI-TPA** (λ_exc_ = 300 nm) and **PEI-Cou** (λ_exc_ = 318 nm) upon addition of an excess (6 equivalents) of ATP and GTP nucleotides. The *y*-axis has been constructed so that the fluorescence of the free ligand is leveled to 0. Titrations were performed in buffered water solution at pH = 7.4, *T* = 298.1 ± 0.1 K.

Determining the exact stoichiometry of the complexes formed between **PEI-TPA** and **PEI-Cou** with ATP and GTP is challenging due to the branched nature of the aminic chains. However, the fluorescent response and the high stability constant, coupled with the degree of aromatic derivatization of the polyamine backbone systems, suggest that **PEI-Cou** can only bind to one nucleotide, whereas **PEI-TPA** can bind to three nucleotides. Figure 7 summarizes the results obtained, showing the percentage of fluorescence change upon the addition of an excess of ATP or GTP to the PEI derivatives. The data clearly demonstrate the high sensitivity of the **PEI-TPA** system for recognizing ATP at physiological pH, with a significant increase in fluorescence (CHEF) upon interaction with ATP and a decrease in fluorescence (CHEQ) for both **PEI-Cou** and **PEI-TPA** when interacting with GTP.

### Fluorescence Lifetime Imaging Microscopy (FLIM) Experiments

**Figure 8:**
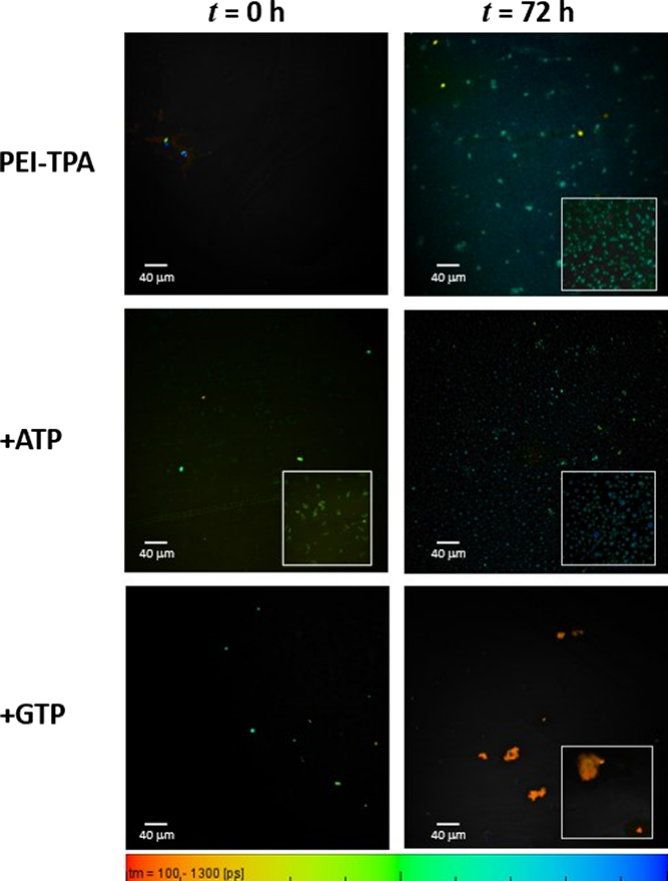
FLIM images (objective 20×, zoom 2×) of **PEI-TPA** in aqueous solution (60 μM, pH = 7.4) and upon addition of ATP and GTP. The images were collected immediately after sample preparation (*t* = 0) and 72 h after. [PEI-TPA] = 60 μM and [ATP] = [GTP] = 3.6 × 10^–4^ M, λ_exc_ = 375 nm. An inset shows selected parts of the FLIM images obtained with objective 40×, zoom 2×. At the bottom, the color scale ruler with the decay time (τ_M_) ranging from 100 to 1300 ps.

Changes in the emission of fluorescence are one of the outputs of the recognition event; however, additional evidence of the detection of the nucleotides comes from the appearance of highly emissive aggregates. Fluorescence lifetime images of **PEI-TPA** in an aqueous solution (60 μM and pH = 7.4) are shown in Figure 8, where FLIM images were obtained for **PEI-TPA**, **PEI-TPA**:ATP, and **PEI-TPA**:GTP at *t* = 0, i.e., immediately after sample preparation, and 72 h after (during 72 h, the samples were left to equilibrate without any stirring). At *t* = 0, it is not possible to observe almost any degree of aggregation in **PEI-TPA (**perhaps only a few compact spots of about 8 μm can be observed). Nevertheless, after 72 h, amorphous aggregates, of roughly 10 μm size, co-exist with smaller-size aggregates. A different behavior can be observed when ATP is added. Six equivalents of ATP highly stimulate the formation of **PEI-TPA** clusters immediately after mixing (see the inset of Figure 8, *t* = 0 h) of an average size of 9 μm (ranging from 4 to 12 μm). Spherical-like structures, of 3–9 μm diameter, are also observed. In the case of the addition of six equivalents of GTP, no evidence of cluster formation is observed, and instead, compact structures (5–6 μm) are formed. These results show that the nucleotide interaction with **PEI-TPA** promotes the formation of aggregates of different structures with distinct photophysical characteristics, as will be discussed below. After 72 h, the 1:6 **PEI-TPA**:ATP system shows the formation of spherical-like structures with an average diameter of 4 μm, whereas the 1:6 **PEI-TPA**:GTP system shows the formation of amorphous aggregates with a broad distribution of sizes. Large aggregates, of 27–30 μm in length, might result from the agglomeration of smaller aggregates.

**Figure 9:**
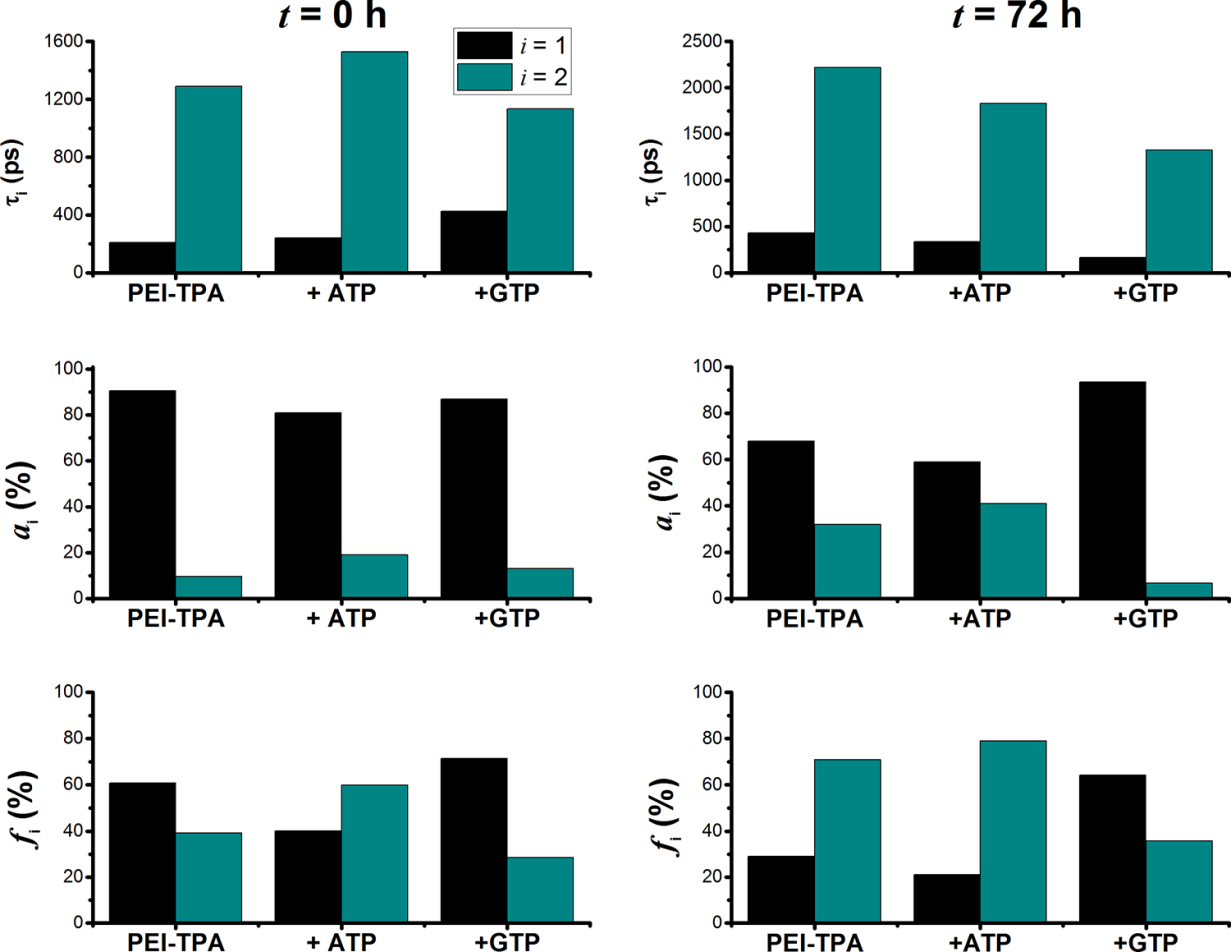
Fluorescence decay times (τ_1_ and τ_2_), pre-exponential factors (*a*_1_ and *a*_2_), and weighted pre-exponential factors  of **PEI-TPA** in buffered aqueous solution (pH = 7.4) and upon addition of ATP and GTP. The left-handed and the right-handed panels correspond to the time-resolved data obtained at *t* = 0 and *t* = 72 h, respectively. [PEI-TPA] = 60 μM and [ATP] = [GTP] = 3.6 × 10^–4^ M, λ_exc_ = 375 nm.

The fluorescence decays of **PEI-TPA** aggregates were fitted with a bi-exponential decay law: 210 ps (90%) and 1290 ps (10%). At *t* = 0 h, upon addition of ATP, the two decay times increase, mirroring the increase of the fluorescence deactivation channel as previously observed (Figures 5 and 7). Moreover, the pre-exponential factor associated with the shorter-lived component, *a*_1_, decreases, whereas the pre-exponential factor associated with the longer-lived component, *a*_2_, increases concomitantly. The same effect is observed in the *f* factor, which mirrors the contribution of each emissive species to the total fluorescence (see Figure 9); the higher *f*_2_ value highlights the fact that τ_2_ is the main emissive component. These observations are in agreement with the CHEF effect observed in the steady-state measurements (Figures 5 and 7). The complex formed upon adding GTP also leads to an increment in the τ_1_ value, but the τ_2_ value decreases; the pre-exponential factors, *a*_1_ and *a*_2_, are only slightly affected by the presence of GTP in the **PEI-TPA** aggregates. Indeed, the main difference occurs in the *f* factors. Comparing the two systems, **PEI-TPA**:ATP and **PEI-TPA**:GTP, *f_i_* increases in the former system, whereas the opposite is observed in the latter.

After 72 h, the effect of *a_i_* and *f_i_* factors becomes more evident in the three systems. Indeed, in the **PEI-TPA** system, *f*_1_ is 30% and the *f*_2_ contribution increases up to 70%. ATP leads to an increment in the contribution of the longer decay component, to the total decay, to 79%, and GTP to a decrease to 35%. These results are in agreement with the findings retrieved from steady-state fluorescence titrations of **PEI-TPA** and **PEI-TPA** with GTP and ATP (Figures 6 and 7).

**Figure 10:**
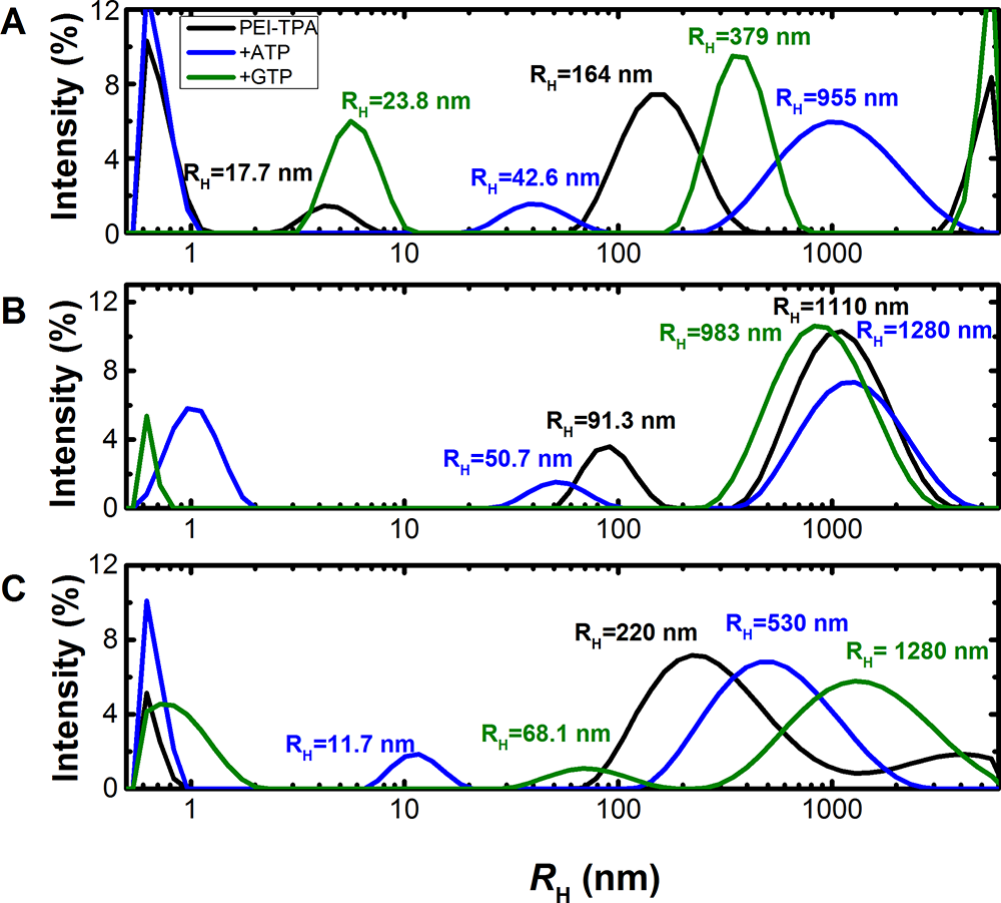
Intensity size distribution curves obtained from DLS of **PEI-TPA** in an aqueous solution (60 μM, pH = 7.4) and upon addition of ATP and GTP (*c* = 3.6 × 10^–4^ M). (a) Immediately after preparation (*t* = 0), (b) after *t* = 3 h, and (c) after *t* = 72 h; *T* = 298 K.

Finally, in order to have a morphological analysis of the aggregates, **PEI-TPA** aqueous solutions (pH = 7.4) were studied at 25 °C, using DLS. The intensity correlation functions, obtained for the **PEI-TPA** solutions, were found to be multiexponential and the obtained relaxation time distributions were multimodal, reflecting the high polydispersity of the studied system. Immediately after the preparation of the samples, it is possible to identify two well-defined modes, attributed to the motion of particles, with apparent hydrodynamic radius (*R*_H_) of 18 and 164 nm (Figure 10 and Table S1). Two additional modes, associated with the motion of particles smaller than 0.3 nm and larger than 6 mm, are also observed. Yet, because of the particle size, and relative contribution, these are not influencing the global behavior and were not further considered. Observation of the DLS size distribution after 3 and 72 h shows that the **PEI-TPA** aggregates grow; indeed, after 3 h, the smaller particles (18 nm) seem to increase to 91 nm and after 72 h, to 220 nm. The larger particles also grow, after 3 h, from 164 to 1100 nm, and now 72 h after, the particle size is now out of our detection limit. Nevertheless, the slower mode seems to have a maximum of around 4 mm. Upon addition of 6 equivalents of ATP or GTP, both **PEI-TPA** modes are shifted, indicating that **PEI-TPA** interacts with both nucleotides originating the formation of larger aggregates. The **PEI-TPA** aggregates formed upon the addition of ATP are larger than with GTP, at *t* = 0. After 72 h, the PEI-TPA/ATP aggregates become smaller than the PEI-TPA/GTP.

## Conclusions

Two polyethylenimine derivatives, namely **PEI-TPA** and **PEI-Cou**, were employed for the selective sensing of GTP and ATP. **PEI-TPA** exhibited fluorescence quenching in the presence of GTP and fluorescence enhancement in the presence of ATP, enabling the differentiation between the two nucleotides. Conversely, **PEI-Cou** displayed a stronger affinity for ATP, forming double hydrogen bonding interactions with the adenine base and exhibiting a binding constant 10 times higher than with GTP. Further investigations using FLIM revealed that the size, shape, and photophysical properties of the aggregates formed by **PEI-TPA** were influenced by the presence of ATP or GTP. In aqueous solution, **PEI-TPA** forms large and amorphous aggregates co-existing with a larger number of smaller aggregates. ATP induced the formation of fluorescent compact spherical-like aggregates with an average diameter of 4 μm. The complexation with GTP led to the formation of large amorphous aggregates, with a disordered structure, which results in a decrease of its photoluminescence. The CHEF vs CHEQ behavior seems to be, respectively, related to the formation of small and compact aggregates vs large and amorphous aggregates, i.e., the morphology of the formed aggregates results from the interaction of **PEI-TPA** with ATP or GTP. These results highlight the potential of PEI derivatives as versatile receptors for nucleotides, offering insights into the biological roles of ATP and GTP and potential applications in disease diagnosis.

## Supporting Information Available

NMR spectra of the PEI backbone and PEI derivatives, percentages of the different amino groups and chemical structure of PEI, spectroscopic changes resulting from the pH modulation or from interaction with ATP and GTP. Morphological analysis of the aggregates performed by DLS (PDF)
