# Supplementary material for: Highly Selective Fluorescent Sensors: Polyethylenimine Derivatives of Triphenylamine and Coumarin for GTP and ATP Interaction via Fluorescence Lifetime Imaging Microscopy
Source: ACS Appl Polym Mater. 2023 Jul 11;5(8):6176–85. doi: 10.1021/acsapm.3c00834 (PMC10426326; doi:10.1021/acsapm.3c00834)
Supplement: Supplementary file 1 — ap3c00834_si_001.pdf [file ap3c00834_si_001.pdf]

Supporting Information

Highly Selective Fluorescent Sensors: Polyethylenimine Derivatives of Triphenylamine and Coumarin for GTP and ATP Interaction via Fluorescence Lifetime Imaging Microscopy

Estefanía Delgado-Pinar[a),b)*], Matilde Medeiros[a)], Telma Costa[a)], J. Sérgio Seixas de Melo[a)]

[a)] University of Coimbra, CQC-IMS, Department of Chemistry, Rua Larga, 3004-535 Coimbra, Portugal.

[b)] Instituto de Ciencia, Molecular, Departamento de Química Inorgánica, Universidad de Valencia, C/Catedrático José Beltrán 2, 46980, Paterna, Spain.

*email: edelgado@qui.uc.pt

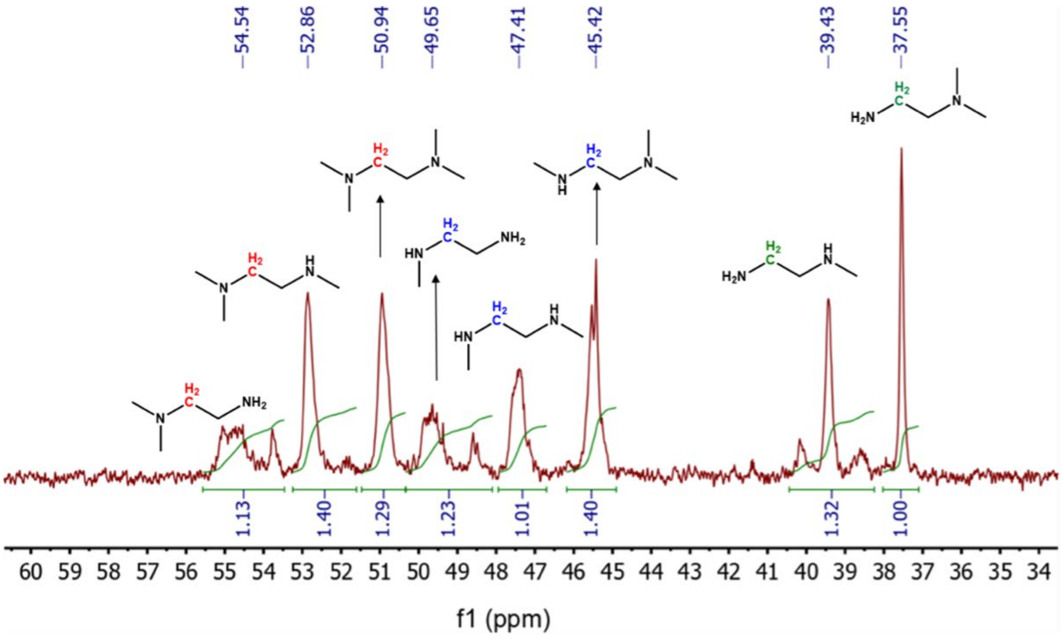

Figure. S1 - ^{13}C inverse-gated spectrum of commercially PEI in D_2O. Structural elements belonging to the different signals: primary (green), secondary (blue) and tertiary (red) are given.

Seq1. Percentages of the different amino groups

The following relations were used for the calculations of the percentages of the amino groups:

$$\% NH_2 = \frac{Area\ from\ primary\ amines}{\left(\frac{Area\ from\ primary\ amines}{3}\right)+\left(\frac{Area\ from\ secondary\ amines}{2}\right)+(Area\ from\ primary\ amines)}$$

$$\% NH = \frac{Area\ from\ secondary\ amines}{\left(\frac{Area\ from\ primary\ amines}{3}\right)+\left(\frac{Area\ from\ secondary\ amines}{2}\right)+(Area\ from\ primary\ amines)}$$

$$\% N = \frac{Area\ from\ tertiary\ amines}{\left(\frac{Area\ from\ primary\ amines}{3}\right)+\left(\frac{Area\ from\ secondary\ amines}{2}\right)+(Area\ from\ primary\ amines)}$$

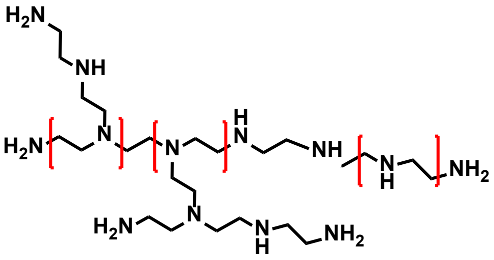

Figure S2. Chemical structure of commercially available branched polyethyleneimines (PEIs). Different building blocks present in the structure are highlighted in red.

Seq2. Quantification of the different amino groups

Number of amine groups on one chain were calculated by dividing molecular weight of PEI by average molecular weight of monomer, i.e 43 (-CH_2-CH_2-NH_2-). As each monomer has one amine group, the no. of monomers was equivalent to no. of amine groups on one PEI chain.

$$Number\ of\ amine\ groups = \frac{Molecular\ weight\ of\ PEI}{Average\ molecular\ weight\ of\ monomer} = \frac{800}{43} = 19$$

What taking into account the percentages results in 8 NH_2 groups, 5 NH groups and 6 N (tertiary) groups

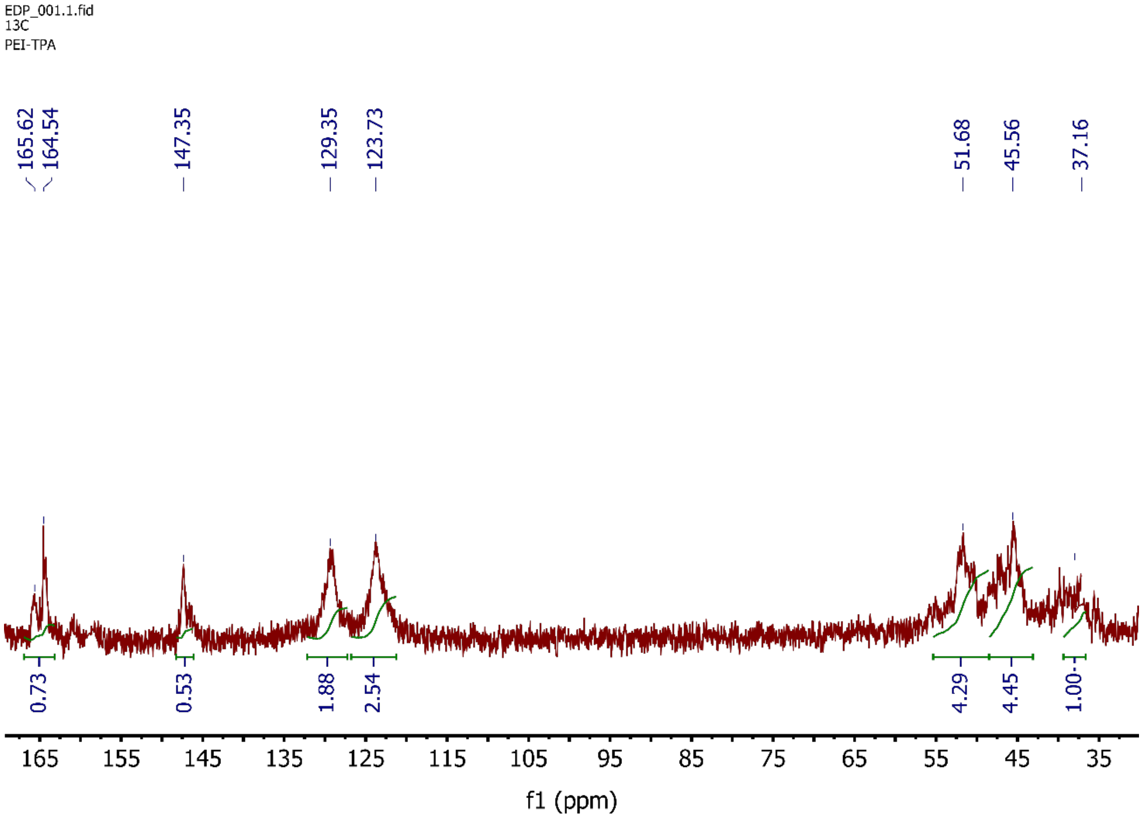

Figure S3. ^{13}C inverse-gated spectrum of **PEI-TPA** in D_2O.

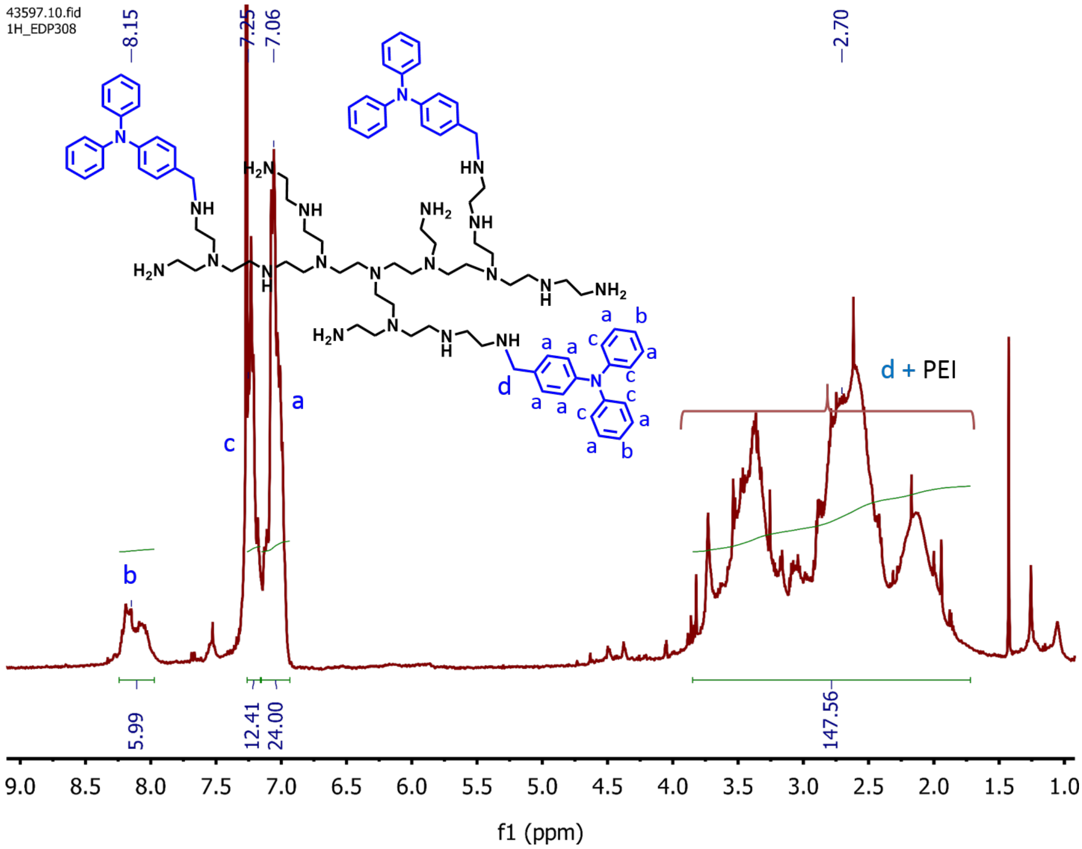

Figure S4. ^{1}H NMR spectrum of **PEI-TPA** in D_2O.

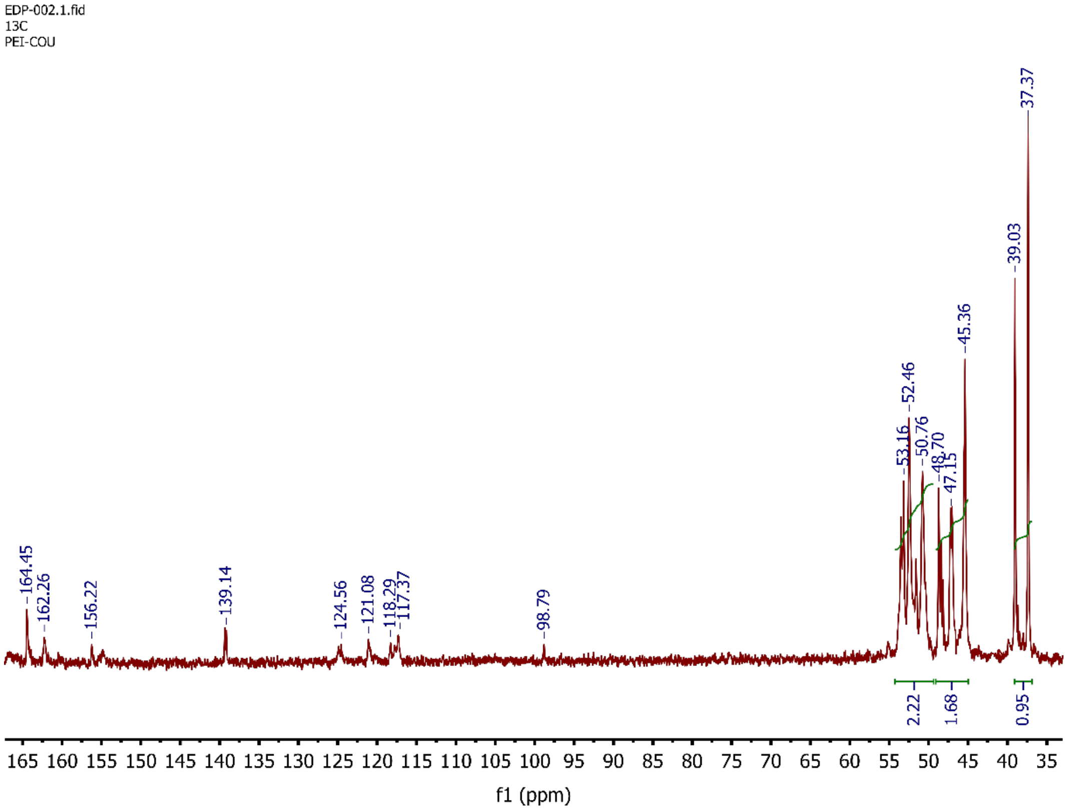

Figure S5. ^{13}C inverse-gated spectrum of **PEI-Cou** in D_2O.

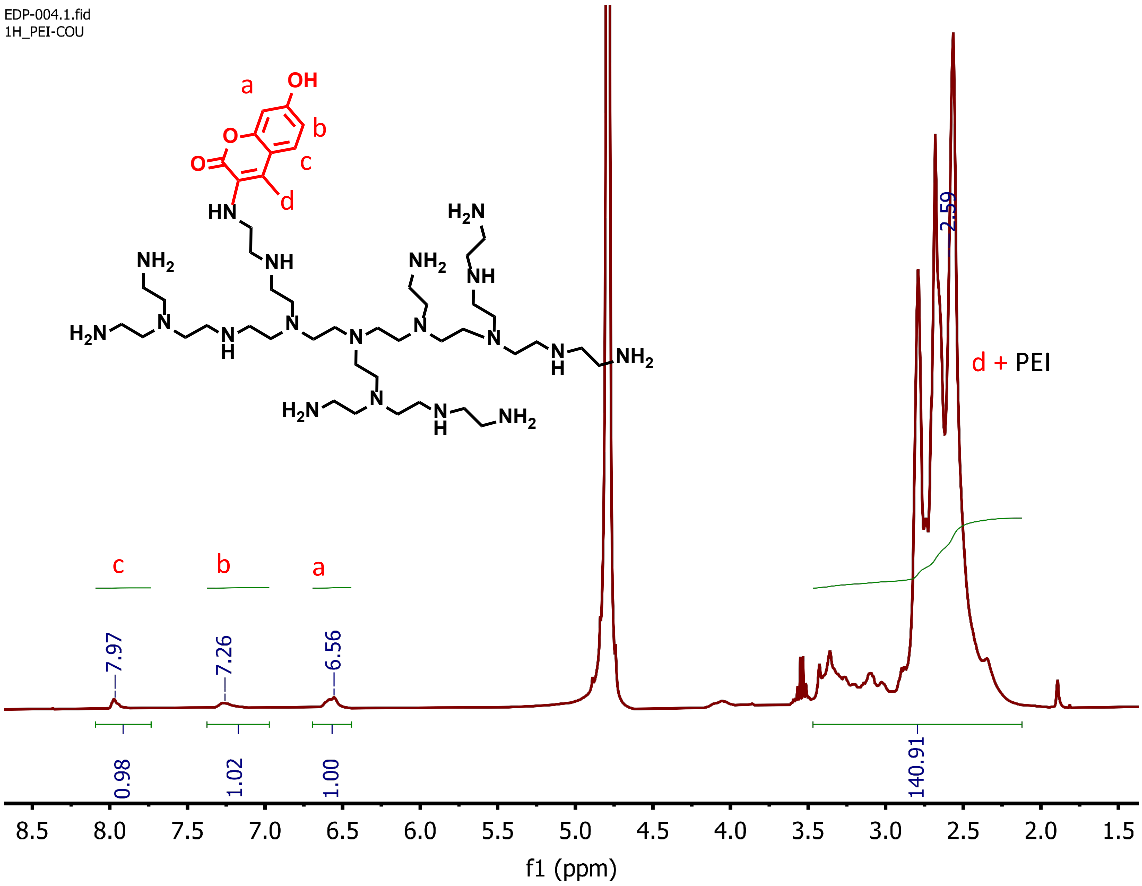

Figure S6. ^{1}H NMR spectrum of **PEI-Cou** in D_2O.

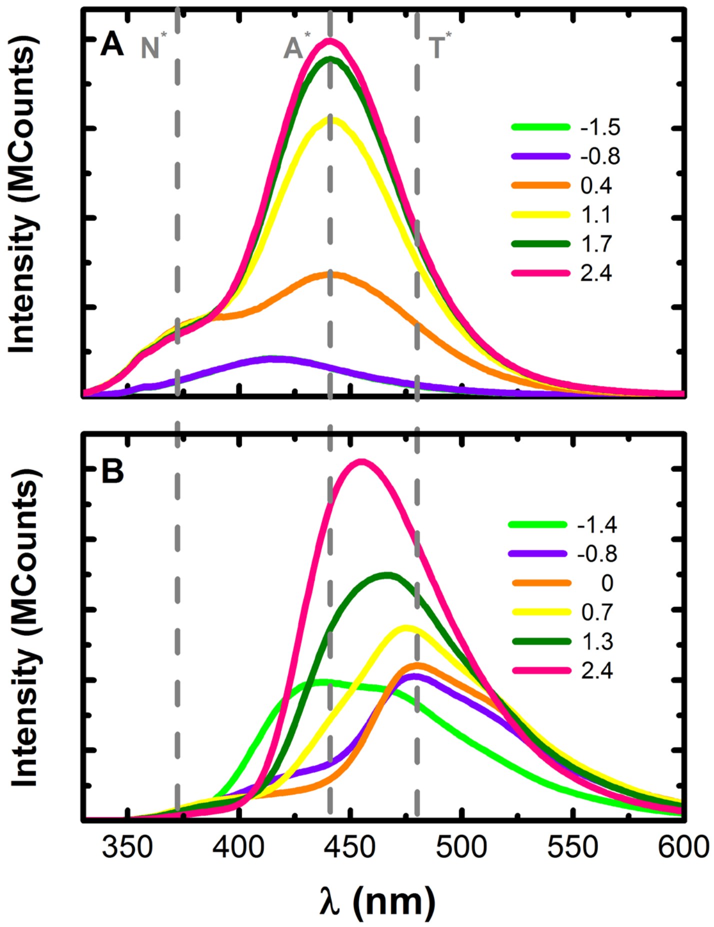

Figure S7. Acidic emission response of A) **PEI-Cou** and B) the coumarin taken as reference, the 7-hydroxy-4-methylcoumarin (7H4MC).

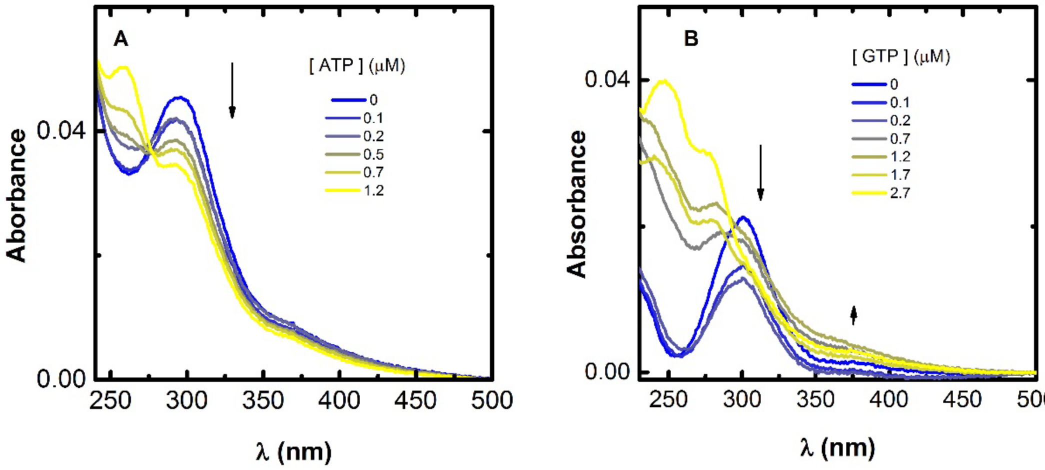

Figure S8. Absorbance spectra resulting from the addition of A) ATP and B) GTP to an aqueous solution of **PEI-TPA** at pH = 7.4. A) [PEI-TPA] = 5.17 x 10^{-7} M and B) [PEI-TPA] = 2.44 x 10^{-7} M. [ATP] = [GTP] from 0 to 6 x 10^{-6} M.

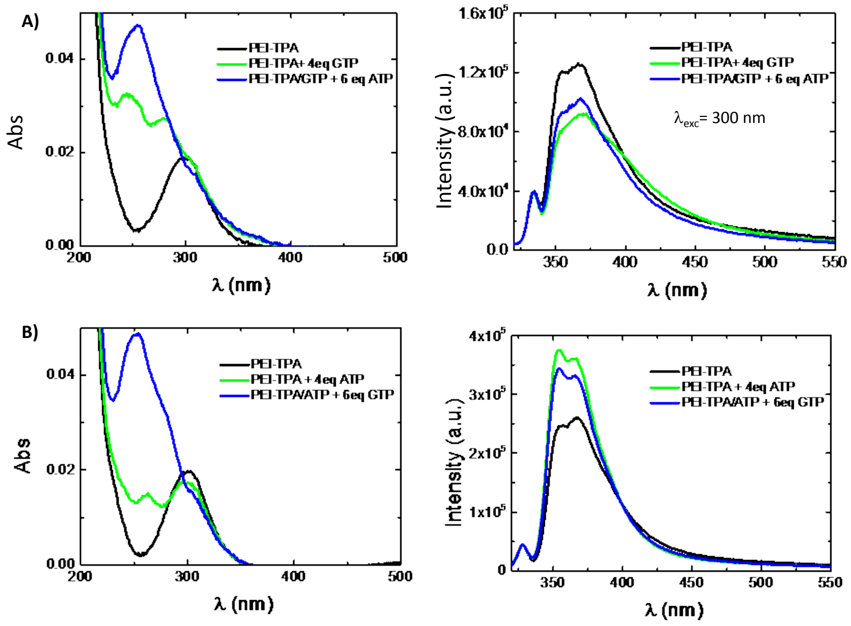

Figure S9. Selectivity of **PEI-TPA** towards the studied nucleotides Absorbance and emission response after the addition of A) GTP versus ATP and B) ATP versus GTP. [**PEI-TPA**] = 2.2 x 10^{-7} M

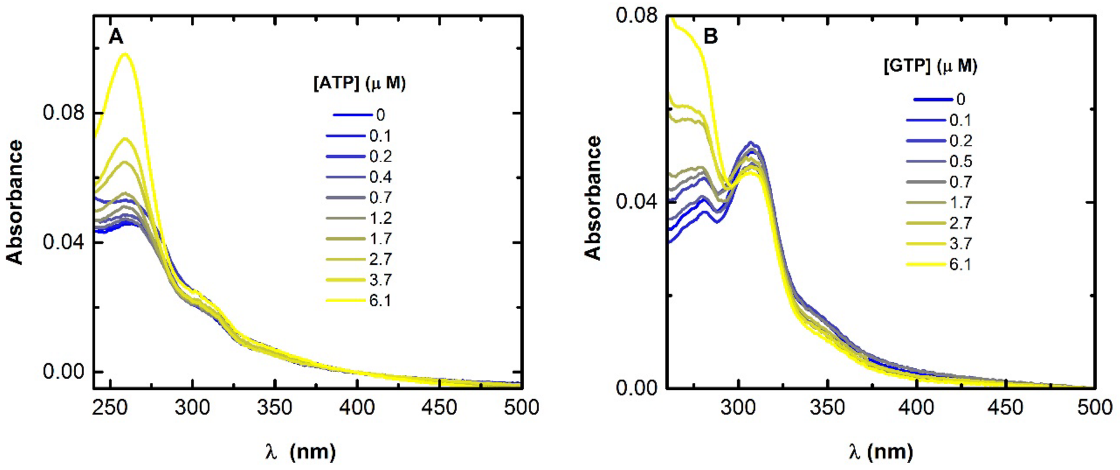

Figure S10. Absorbance spectra resulting from the addition of A) ATP and B) GTP to an aqueous solution of **PEI-Cou** at pH = 7.4. A) [PEI-Cou] = 3.81 x 10^{-6} M and B) [PEI-Cou] = 8.86 x 10^{-6} M. [ATP] = [GTP] from 0 to 6 x 10^{-6} M.

Table S1. Hydrodynamic radius of **PEI-TPA** in aqueous solution (60 μM, pH = 7.4), and upon addition of ATP and GTP (c = 3.6 × 10^{-4} M), immediately after preparation (t=0) and 3 h and 72h after, at 25 ºC.

System	PEI-TPA	+ATP	+GTP
t = 0	18	43	24
	164	955	379
t = 3h	---	7.5	---
	91	51	---
	1110	1280	983
t = 72h	---	12	68
	220	530	1280
